# Hyperoxia exposure induces ferroptosis and apoptosis by downregulating PLAGL2 and repressing HIF-1α/VEGF signaling pathway in newborn alveolar typeII epithelial cell

**DOI:** 10.1080/13510002.2024.2387465

**Published:** 2024-08-05

**Authors:** Yuting Zhu, Hongmei Hou, Yawen Li, Yanyu Zhang, Yuanyuan Fang, Si Chen, Le Zhang, Weilai Jin, Yahui Zhou

**Affiliations:** Department of Neonatology, The Affiliated Children’s Hospital of Jiangnan University, Wuxi, People’s Republic of China

**Keywords:** Bronchopulmonary dysplasia (BPD), PLAGL 2, HIF-1α/VEGF pathway, lung injury

## Abstract

**Backgroud:**

Bronchopulmonary dysplasia (BPD) is one of the most important complications plaguing neonates and can lead to a variety of sequelae. the ability of the HIF-1α/VEGF signaling pathway to promote angiogenesis has an important role in neonatal lung development.

**Method:**

Newborn rats were exposed to 85% oxygen. The effects of hyperoxia exposure on Pleomorphic Adenoma Gene like-2 (PLAGL2) and the HIF-1α/VEGF pathway in rats lung tissue were assessed through immunofluorescence and Western Blot analysis. In cell experiments, PLAGL2 was upregulated, and the effects of hyperoxia and PLAGL2 on cell viability were evaluated using scratch assays, CCK-8 assays, and EDU staining. The role of upregulated PLAGL2 in the HIF-1α/VEGF pathway was determined by Western Blot and RT–PCR. Apoptosis and ferroptosis effects were determined through flow cytometry and viability assays.

**Results:**

Compared with the control group, the expression levels of PLAGL2, HIF-1α, VEGF, and SPC in lung tissues after 3, 7, and 14 days of hyperoxia exposure were all decreased. Furthermore, hyperoxia also inhibited the proliferation and motility of type II alveolar epithelial cells (AECII) and induced apoptosis in AECII. Upregulation of PLAGL2 restored the proliferation and motility of AECII and suppressed cell apoptosis and ferroptosis, while the HIF-1α/VEGF signaling pathway was also revived.

**Conclusions:**

We confirmed the positive role of PLAGL2 and HIF-1α/VEGF signaling pathway in promoting BPD in hyperoxia conditions, and provided a promising therapeutic targets.

## Introduction

In 1967, Northway et al. first used ‘bronchopulmonary dysplasia’ (BPD) to describe lung injury caused by oxygen and mechanical ventilation in newborns [1]. In 2019, BPD was redefined to classify the severity of the disease based on the type of respiratory support at 36 weeks postmenstrual age, regardless of the use of supplemental oxygen [2]. Despite advances in neonatal intensive care over the past half century, BPD remains a major complication in premature infants, with over 40% of premature infants experiencing varying degrees of BPD, and the severity of BPD being inversely related to gestational age [3, 4]. The occurrence of BPD is a complex process, and severe BPD may lead to certain sequelae, such as decreased lung function, asthma, and pulmonary hypertension [5–7]. In addition to prematurity, prenatal factors such as maternal smoking history and socio-economic status are associated with the development of BPD in newborns. Furthermore, in terms of genetics, the excessive transmission of genes such as angiotensin-converting enzyme from parents also increases the risk of BPD [6]. Within the first few days of a newborn's life, the concentration of oxygen can affect the development of the alveoli, and high environmental oxygen concentrations can lead to alveolar damage, resulting in BPD, as the body's antioxidant enzyme system is not fully developed [8].

Hypoxia-inducible factor-1α (HIF-1α) and vascular endothelial growth factor (VEGF) are important regulatory factors for pulmonary microvascular formation and alveolar development, playing a critical role in the pathogenesis of BPD [9–11]. HIF-1α and VEGF are closely related to the concentration of oxygen. Recent studies have shown that their expression levels and functions are restricted when exposed to high levels of oxygen [12, 13]. The expression of HIF-1α is initially imbalanced under hyperoxia exposure. VEGF is closely related to pulmonary vascular development, and as a downstream signaling molecule of HIF-1α, its expression level is positively correlated with HIF-1α. When VEGF is inhibited, both vascular formation and alveolar development in newborn rats lungs are weakened [14, 15].

Pleomorphic Adenoma Gene like-2 (PLAGL2) is a zinc finger protein that belongs to the PLAG zinc finger protein subfamily, along with PLAG1 and PLAGL1 [16]. As an important transcriptional factor, PLAGL2 regulates various cancer processes through different signaling pathways, including hepatocellular carcinoma, gastric cancer, bladder cancer. [17–19]. PLAGL2 can activate USP37, which in turn acts on Snail 1 to prevent its ubiquitination and promote gastric cancer cell proliferation and metastasis [18]. As an upstream signaling molecule in hepatocellular carcinoma, PLAGL2 can regulate the expression of HIF-1α. Silencing PLAGL2 leads to a downregulation of HIF-1α expression in hepatocellular carcinoma [20]. Recent studies have shown that the expression of PLAGL2 is significantly correlated with oxygen concentration, and its expression is upregulated in response to hypoxia [21]. This suggests that PLAGL2 may potentially serve as an upstream regulatory factor of HIF-1α, participating in the pathogenesis of BPD during hyperoxia exposure.

In this study, we investigated the role of PLAGL2 and the HIF-1α/VEGF signaling pathway in the process of BPD. The results showed that upregulating PLAGL2 could alleviate pulmonary damage, reduce apoptosis, and inhibit the development of BPD by reactivating the HIF-1α/VEGF signaling pathway that had been suppressed by hyperoxia. This study provides theoretical support for PLAGL2 as a potential therapeutic target for BPD.

## Results

### When exposed to hyperoxia, the PLAGL2 and HIF-1α/VEGF signaling pathways in lung tissue are suppressed

**Figure 1.**
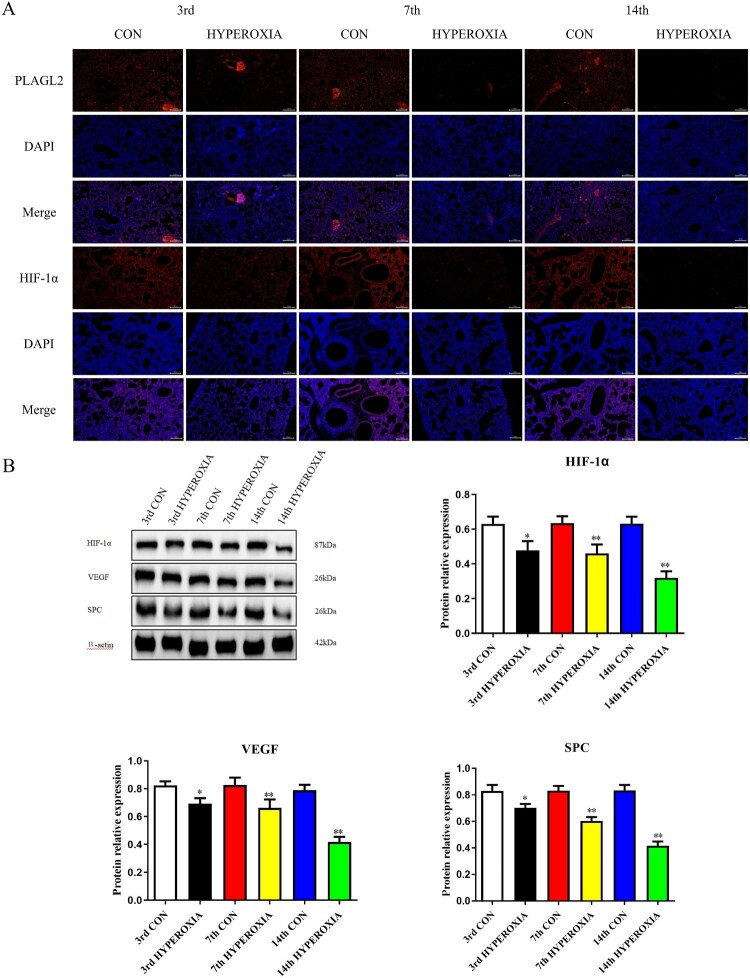
Hyperoxia exposure reduces the expression of PLAGL2, HIF-1α, VEGF, and SPC. (A) Immunofluorescence staining revealed that the expression of PLAGL2 and HIF-1α in lung tissues of the CON group was higher compared to the corresponding HYPEROXIA group on the 3rd day, 7th day, and 14th day. (B) Western blot analysis was performed to assess the protein expression levels of HIF-1α, VEGF, and SPC. The protein expression levels of HIF-1α, VEGF, and SPC were significantly decreased after hyperxoia exposure compared to the CON group. Statistical significance was determined as *p < 0.05, **p < 0.01, ***p < 0.001.

Immunofluorescence results indicate that the expression levels of PLAGL2 and HIF-1α in rats lung tissue decrease after hyperoxia exposure, while their expression levels in the control group remain relatively stable. Furthermore, as the duration of rats exposure to hyperoxia increases, the degree of inhibition of PLAGL2 and HIF-1α expression also deepens (Figure 1A). Western blot analysis also shows that the expression of HIF-1α significantly decreases after 3 days of hyperoxia exposure (*p *< 0.05), and it is greatly inhibited at day 7 and day 14 (*p *< 0.01). The expression of VEGF and SPC in lung tissue is also suppressed by hyperoxia, with a significant decrease at day 3 (*p *< 0.05) and a remarkable inhibition at day 7 and day 14 (*p *< 0.01) (Figure 1B).

### Upregulation of PLAGL2 alleviates AECII migration and proliferation inhibition caused by hyperoxia exposure

**Figure 2.**
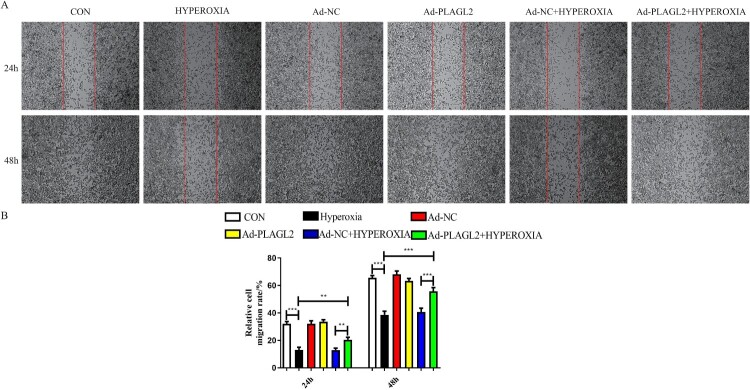
Upregulation of PLAGL2 can restore the migration ability of AECII cells inhibited by hyperoxia. (A)Representative images of wound healing assay for ACEII cells at 24 and 48 h. Red lines indicate the leading edge of the migrating cells (magnification × 40). (B)Migration of ACEII cells was tested and the distance of the wound was calculated up to 48 h. Compared with CON group. *p < 0. 05, **p < 0. 01, ***p < 0. 001 was considered statistically significant.

Scratch assay results showed that the migratory capacity of rats AECII was significantly inhibited after 24 and 48 h of hyperoxia exposure (*p *< 0.001). After upregulating PLAGL2, a partial recovery of the inhibitory effect on AECII migration was observed (*p *< 0.01). It is worth noting that the upregulation of PLAGL2 under non-hyperoxia conditions did not significantly affect AECII migration (*p *> 0.05) (Figure 2).

**Figure 3.**
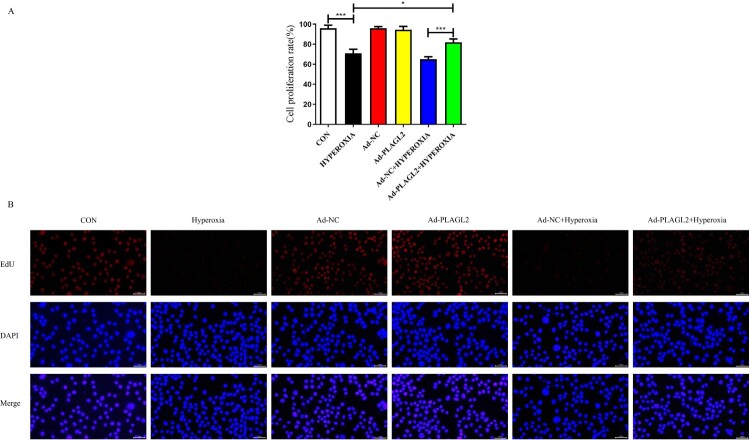
demonstrates that upregulation of PLAGL2 can attenuate the inhibitory effect of hyperoxia on AECII cell proliferation. (A) CCK-8 assay showed that the proliferation capacity was restored in the Ad – PLAGL2 + HYPEROXIA group compared to the HYPEROXIA group. (B) EDU assay was performed to assess cell proliferation, and it revealed enhanced proliferation ability of AECII cells after upregulating PLAGL2. Statistical significance was determined as *p < 0.05, **p < 0.01, ***p < 0.001.

We used CCK-8 and EdU assays to evaluate the proliferation capacity of AECII. The proliferation ability of AECII was significantly inhibited under hyperoxia conditions (*p *< 0.001). We hypothesized that upregulation of PLAGL2 could alleviate the inhibitory effect of hyperoxia on cell proliferation. The CCK-8 results showed that the proliferation rate in the Ad – PLAGL2 + HYPEROXIA group was significantly higher than that in the HYPEROXIA group (*p *< 0.05) and the Ad-NC + HYPEROXIA group (*p *< 0.001) (Figure 3A). This hypothesis was further supported by the results of the EdU assay, which showed that upregulation of PLAGL2 partially restored the suppressed proliferation ability of AECII cells due to hyperoxia exposure, indicating a positive role of PLAGL2 in AECII cell proliferation. Interestingly, similar to the scratch assay, there was no significant difference in the proliferation rate between the Ad-PLAGL2 group and the Ad-NC group, suggesting that upregulation of PLAGL2 under non-hyperoxia exposure conditions did not significantly affect AECII proliferation ability (*p *> 0.05) (Figure 3B).

### Upregulation of PLAGL2 activates the HIF-1α/VEGF signaling pathway in AECII

**Figure 4.**
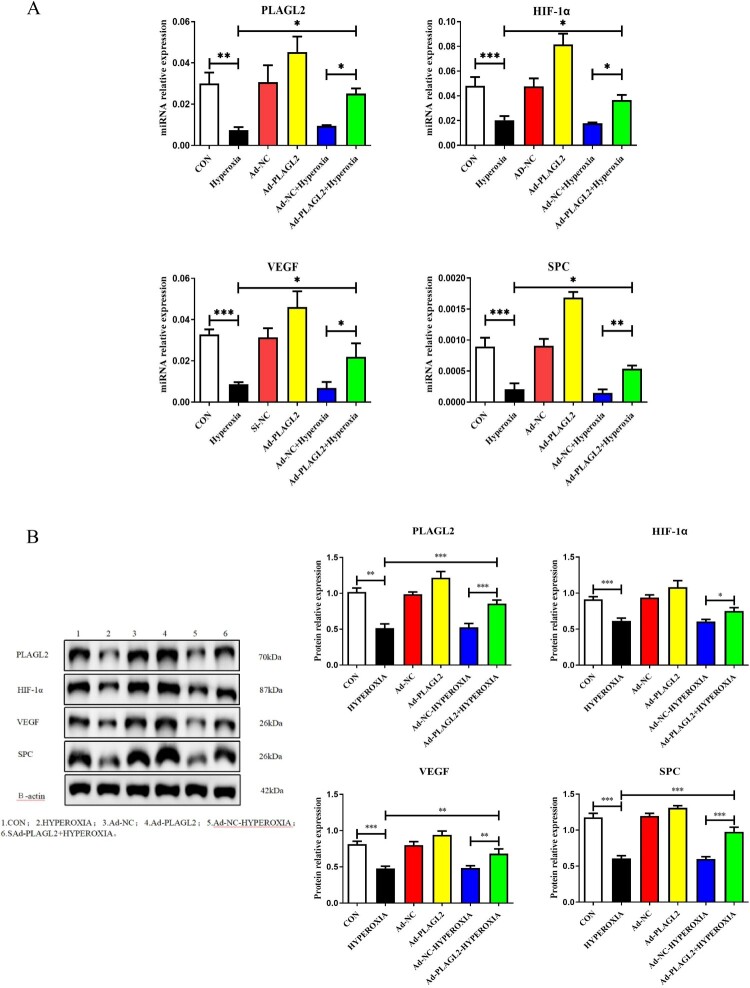
Upregulation of PLAGL2 activates the HIF-1α/VEGF signaling pathway in AECII cells. (A) Upregulation of PLAGL2 increased the mRNA expression of genes related to the HIF-1α/VEGF signaling pathway. (B) Western blot analysis revealed that upregulation of PLAGL2 enhanced the protein expression of the HIF-1α/VEGF signaling pathway. Statistical significance was determined as *p < 0.05, **p < 0.01, ***p < 0.001.

Real-time PCR results showed that the mRNA expression levels of PLAGL2 (*p *< 0.01), HIF-1α (*p *< 0.001), VEGF (*p *< 0.001), and SPC (*p *< 0.001) were decreased after 24 h of hyperoxia exposure. Compared to the HYPEROXIA group, the relative mRNA expression levels of PLAGL2, HIF-1α, VEGF, and SPC were significantly increased in the Ad – PLAGL2 + HYPEROXIA group (*p *< 0.05) (Figure 4A). Western blot was used to investigate the effect of upregulated PLAGL2 on the HIF-1α/VEGF signaling pathway. Consistently with the RT–PCR results, compared to the CON group, the protein expression levels of PLAGL2 (*p *< 0.01), HIF-1α (*p *< 0.001), VEGF (*p *< 0.001), and SPC (*p *< 0.001) were all suppressed in the HYPEROXIA group after hyperoxia treatment. Compared to the HYPEROXIA group, the protein expression levels of PLAGL2 (*p *< 0.001), VEGF (*p *< 0.01), and SPC (*p *< 0.001) were significantly increased in the Ad – PLAGL2 + HYPEROXIA group. It is worth noting that although the protein expression level of HIF-1α increased, the result was not significant (*p *> 0.05) (Figure 4B).

### Upregulation of PLAGL2 alleviates apoptosis in AECII

**Figure 5.**
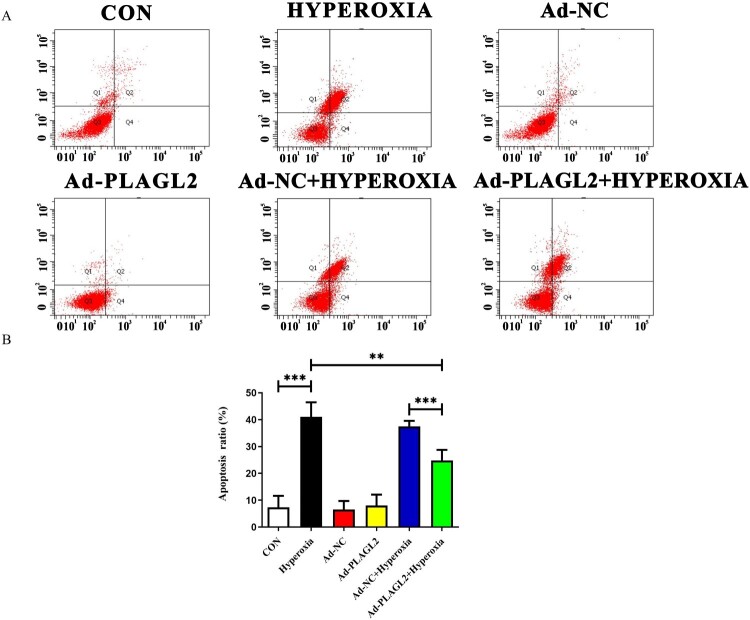
Flow cytometry detects apoptosis in AECII cells. (A) Upregulation of PLAGL2 can alleviate hyperoxia-induced apoptosis in AECII cells. (B)Quantitative analysis of apoptotic cell percentages. Percentage of cell apoptosis = Q2 (early apoptosis) + Q4 (late apoptosis). *p < 0. 05, **p < 0. 01, ***p < 0. 001 was considered statistically significant.

We used Annexin V-FITC/PI double staining to detect apoptosis in AECII (Figure 5A). The apoptotic rate was calculated as Q2 (late apoptotic rate) plus Q4 (early apoptotic rate). Compared to the CON group, the apoptotic rate of AECII significantly increased after hyperoxia exposure (*p *< 0.001) (Figure 5B). The apoptotic rate in the Ad-PLAGL2 + HYPEROXIA group was lower than that in the HYPEROXIA group (*P* < 0.05), indicating that upregulation of PLAGL2 can protect AECII and reduce apoptosis occurrence.

### Upregulation of PLAGL2 alleviates ferroptosis in AECII

**Figure 6.**
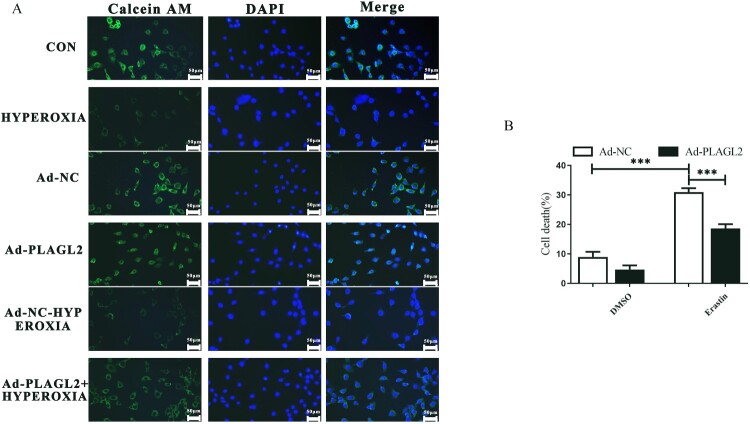
PLAGL2 inhibits ferroptosis in AECII cells. (A) AECII cells were stained using the Calcein AM staining kit. (B) Ad-NC and Ad-PLAGL2 AECII cells were treated with DMSO and erastin for 8 hours. Cell viability was examined through Trypan blue staining. *p < 0. 05, **p < 0. 01, ***p < 0. 001 was considered statistically significant.

To determine whether upregulation of PLAGL2 regulates cell ferroptosis, we first stained the AECII cells with Calcein AM and then observed the fluorescence intensity under a fluorescence microscope. Calcein itself is a metal chelator, and its fluorescence signal is quenched when it complexes with iron ions under physiological conditions. Compared to the control group, the intracellular iron content significantly increased after high oxygen treatment. Upregulation of PLAGL2 can reduce the intracellular iron concentration elevated due to hyperoxia (Figure 6A). Meanwhile, we also treated AECII cells and Ad-PLAGL2 AECII cells with the ferroptosis inducer erastin. The results showed that the cell viability of erastin-treated AECII cells significantly decreased. However, overexpression of PLAGL2 inhibited ferroptosis and restored the cell viability of erastin-treated AECII cells. Overall, PLAGL2 has an inhibitory effect on ferroptosis in AECII cells. (Figure 6B).

## Discussion

When the fetal lungs are damaged in the late stage of development, a common chronic lung disease called BPD can occur. The causes of BPD include numerous factors such as prenatal, postnatal, and genetic factors. During the later stages of lung development, the alveoli develop from their initial form into type I and type II epithelial cells, with the latter having an enhanced ability to secrete surfactant. This is followed by a marked expansion of the pulmonary vasculature, with the formation of new arteries and veins and the creation of an infantile air-blood barrier. Surface-active substances are also crucial in the later stages of lung development, reducing alveolar surface tension and helping the alveoli to expand without collapsing, which contributes to alveolar stability [22, 23]. During pregnancy, the uterus is in a relatively hypoxic environment, and the fetal antioxidant system is not fully developed, so the fetus is very sensitive to changes in oxygen concentration in the environment. In the hyperoxia exposure model, pulmonary microvasculature, alveoli and surface active substances are affected to different degrees [24, 25]. Therefore, in this experiment, we treated rats with 85% O_2_ and executed them immediately after treatment to simulate BPD using a hyperoxia-induced lung injury model to find the mechanism of its development, which can contribute to the clinical prevention and treatment of BPD.

It has been reported that activation of the HIF-1α/VEGF signaling pathway can alleviate retinal complications caused by subarachnoid hemorrhage [26]. In a stroke model, astrocytes promote angiogenesis and facilitate neurological recovery by activating the HIF-1α/VEGF signaling pathway [27]. HIF-1α is involved in fetal lung development and plays an important transcriptional regulatory role in fetal vascular development under hypoxic conditions. Furthermore, the development of pulmonary blood vessels is crucial for alveolar formation [28, 29]. Previous studies have shown that the expression level of HIF-1α in the lungs of newborns decreases after hyperoxia exposure. Therefore, the loss of HIF-1α expression may be one of the important reasons for the occurrence of bronchopulmonary dysplasia (BPD) in premature infants [30]. In our experiment, we also found that the protein expression level of HIF-1α in lung tissue significantly decreased after 7 and 14 days of 85% O2 treatment. In cell experiments, the upregulation of PLAGL2 partially reversed the inhibition of HIF-1α expression caused by hyperoxia exposure. Vascular endothelial growth factor (VEGF) plays an important role in promoting angiogenesis. It induces endothelial cell division, inhibits apoptosis, and is a major downstream gene of HIF-1α in angiogenesis. The expression level of VEGF also significantly decreased after 7 and 14 days of hyperoxia exposure, affecting the development of microvessels and alveoli in premature infants.

According to reports, inhibiting ferroptosis can alleviate the damage of bronchopulmonary dysplasia (BPD). Deferoxamine is capable of decreasing iron ion levels, inhibiting the activity of prolyl-4-hydroxylases, and improving alveolar formation in hyperoxia exposed neonatal rats [10, 29]. ETS1 plays a protective role in bronchopulmonary dysplasia (BPD) by inhibiting oxidative stress-induced ferroptosis [31]. To investigate the impact of PLAGL2 on bronchopulmonary dysplasia (BPD), we established an AECII cell line with upregulated PLAGL2 expression. The results revealed that AECII cell proliferation and motility were suppressed following exposure to hyperoxia, but upregulation of PLAGL2 could alleviate these damages induced by hyperoxia. SPC enhances the absorption and diffusion of pulmonary surfactant lipids on the alveolar surface, maintaining the normal morphology of alveoli and participating in respiratory movements. Upregulation of PLAGL2 can activate the HIF-1α/VEGF signaling pathway that is suppressed due to high oxygen exposure, reducing apoptosis. This is not only related to the angiogenic activity of VEGF but may also depend on the protective effect of SPC on the pulmonary homeostasis. After hyperoxia exposure, there was a significant increase in intracellular iron ion levels in AECII cells, while overexpression of PLAGL2 attenuated this change. Furthermore, to further demonstrate the inhibitory effect of PLAGL2 on ferroptosis, we simultaneously treated PLAGL2-upregulated AECII cells with the ferroptosis inducer erastin. The results showed that upregulation of PLAGL2 could reduce the decrease in cell viability caused by ferroptosis.

Recent studies have shown that PLAGL2 can regulate the expression level of HIF-1α, and our experiments have verified this result. Moreover, in a BPD model induced by hyperoxia, we found that upregulating PLAGL2 could affect the HIF-1α/VEGF signaling pathway to alleviate the damage caused by hyperoxia and reduce BPD. However, we have not determined whether the relief of BPD by the HIF-1α/VEGF signaling pathway is due to promoting neovascularization. In future studies, further investigations are needed to deepen our understanding of the role of the HIF-1α/VEGF signaling pathway in the mechanism of BPD.

## Conclusions

**Figure 7.**
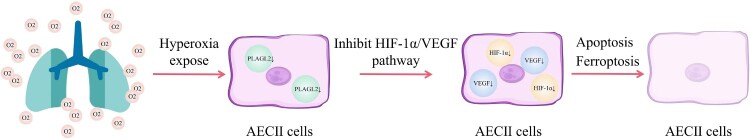
Exposure to hyperoxia inhibits the expression levels of PLAGL2 in AECII cells, which in turn suppresses the HIF-1α/VEGF signaling pathway and leads to cellular apoptosis and ferroptosis.

In conclusion, the findings indicate that PLAGL2 plays a protective role in mitigating lung damage induced by excessive oxygen exposure through the activation of the HIF-1α/VEGF signaling pathway, leading to a reduction in apoptosis and ferroptosis, ultimately alleviating BPD. PLAGL2 holds potential as a novel therapeutic target for the treatment of BPD (Figure 7).

## Materials and methods

### Animals and hyperoxia exposure

36 neonatal rats were randomly divided into hyperoxia group (exposed to 85% O_2_ environment from birth to the end of the experiment) and control group (normal oxygen, 21% O_2_). The animals had free access to water and food during the experiment. At 3, 7, and 14 days, 6 rats were selected from each group, anesthetized with pentobarbital sodium and lung tissue was collected. The left lung was fixed in paraformaldehyde (PFA) and subjected to immunofluorescence analysis, while the right lung tissues of rats were subjected to Western Blot analysis.

### Reagents and antibodies

PLAGL2 primary antibodies (ab139509, abcam, USA), HIF-1α primary antibodies (ab179483, abcam, USA),VEGF primary antibodies (251622, ZENBIO Biotech, Chengdu, China), SPC primary antibodies (10774-1-AP, Proteintech, USA), fluoresceine isothiocyanate (FITC) labeled goat anti-rabbit fluorescent secondary antibody (Bioss, Beijing, China), RIPA Lysis Buffer(Cell Signaling Technology, MA,USA); BCA Protein Assay Kit(Vazyme, Nanjing, China),polyvinylidene difluoride (PVDF) membranes (Millipore, Burlington, USA); GAPDH primary antibody (Goodhere Biotech, Hangzhou, China); PCR primers (Genscript Biotech, Nanjing, China),rat AECII cells (Bnbio, Beijing, China); EdU Cell Proliferation Kit(Beyotime, Shanghai, China);Annexin V-fluorescein isothiocyanate (FITC)/propidium iodide (PI) apoptosis detection kit and Cell Counting Kit-8 (Vazyme, Nanjing, China); Trypan blue(Beyotime, Shanghai, China); Calcein AM staining kit (Beyotime, Shanghai, China); cDNA synthesis kit (Takara, Beijing, China); Trizol reagent (Invitrogen, Shanghai, China).

### Immunofluorescence

The fixed lung tissue was dehydrated and embedded in paraffin to obtain 4-μm-thick lung tissue sections. After deparaffinization and hydration, the sections were incubated with sodium citrate at 110°C for 10 minutes. The tissue sections and cell coverslips were washed three times with PBS, followed by incubation in blocking solution for 1 hour. After three washes with PBS, the tissue sections were incubated with primary antibodies against PLAGL2 (dilution 1:200) and HIF-1α (dilution 1:200). Incubate the sliced sample with the corresponding secondary antibody, FITC-labeled goat anti-rabbit IgG, at 25°C for 1 hour. Finally, DAPI was used to identify cell nuclei.

The observers were blind to the grouping, and three tissue sections were selected from each group. Five random areas were observed at ×200 magnification under a fluorescence microscope at the same parameters (brightness, contrast, and LUT). Sholl analysis was performed using Image Pro Plus 6.0.2.4 Western blot analysis.

### Western blot analysis

Total protein was isolated from lung tissue using radioimmunoprecipitation assay (RIPA) and quantified using the bicinchoninic acid assay (BCA) protein detection kit. The isolated protein was diluted in loading buffer, boiled for 5 min, and then separated by 12.5% SDS-PAGE using 20 μg of protein sample. The separated proteins were transferred onto a polyvinylidene fluoride (PVDF) membrane. The membrane was probed with primary antibodies against PLAGL2 (dilution 1:1000), HIF-1α (dilution 1:1000), VEGF (dilution 1:1000), and SPC (dilution 1:1000) at 4°C overnight, followed by incubation with horseradish peroxidase-conjugated secondary antibodies at room temperature for 2 hours. The protein bands were detected using an ECL advanced system (Millipore) and quantified using Photoshop software.

### Real-time PCR analysis

Total RNA from tissues or cells were extracted using Trizol reagent (Invitrogen, cat number: 15596026), and reverse transcribed into cDNA using a cDNA synthesis kit (Takara, cat number: RR047A). Quantitative PCR was done with a Step one plus Real-Time PCR system (Applied Biosystems, USA) with gene-specific primers. The amount of RNA was calculated using the comparative threshold cycle method. All primers were custom-made by Genscript. The primer sequences are shown in Table 1.

**Table 1.** Sequences of the real-time PCR primers.

Gene	Primer sequences
HIF-1α-F	5'-ACGATTGTGAAGTTAATGCT-3'
HIF-1α-R	5'-AACCAACAGAAACGAAACCCC-3'
VEGF-F	5'-GGGCAAAGTGAGTGACCTG-3'
VEGF-R	5'-CAGCCCAGAAGTTGGACGA-3'
SPC-F	5'-CCTTGAGATGAGCATCGGAG-3'
SPC-R	5'-AGAAGGTAGCGATGGTGTCT-3'
GAPDH-F	5' – CAAGTTCAACGGCACAGTCAAG – 3'
GAPDH-R	5' – ACATACTCAGCACCAGCATCAC – 3'

### Cell lines, cell transfection and hyperoxia treatment

Rat alveolar type II epithelial cells (AEC II) were cultured in RPMI-1640 medium at 37°C in a 5% CO_2_ environment. The cell culture medium contained 10% (v/v) fetal bovine serum, 100 U/mL penicillin, and 100 μg/mL streptomycin. There were six groups of AEC II cells, including CON (AEC II cell group), HYPEROXIA (AEC II cell group with hyperoxia exposure), Ad-NC (negative control group), Ad-PLAGL2 (AEC II cell group with PLAGL2 upregulation), Ad-NC-HYPEROXIA (negative control group with hyperoxia exposure), and Ad-PLAGL2 + HYPEROXIA (AEC II cell group with PLAGL2 upregulation and hyperoxia exposure). When the fusion rate of AEC II cells reached 70%, they were treated with Lipofectamine 3000 and PLAGL2 plasmid, and then cultured in serum-free medium for 6 hours. The cell culture plates were then placed in a sterile module incubator (MIC-101, Billups-Rothenberg Inc., Del Mar, CA) and exposed to hyperoxia (85% O_2_ and 5% CO_2_) for 24 hours.

### Cell proliferation assay

A suspension of alveolar type II (AEC II) cells was cultured to logarithmic growth phase and 100 μL PBS was added around each well to prevent liquid evaporation in the experimental wells and affecting the OD value. After the exposure time, 10 μL of CCK-8 detection reagent was added to each well under light avoidance conditions. The 96-well plate was gently shaken and incubated for another 1 hour in the incubator. Then, the absorbance value at 450 nm was measured using a microplate reader (Shanghai Flash, China). In addition, the EdU Cell Proliferation Assay Kit was used to perform the EdU assay. After incubating with EdU solution, AEC II cells were fixed with 4% paraformaldehyde and stained with the Click-IT reaction cocktail. Finally, images were captured using a fluorescence microscope (Zeiss, Germany).

### Scratch assay

Four horizontal lines were evenly drawn on the bottom of a 6-well plate in advance for subsequent photo taking and positioning. After the cells in the well grew to testable level, a line was drawn vertically and moderately with a 200 μL pipette tip in the well. The plate was then washed three times with PBS to remove any cells that had fallen off due to the scratch. Images were taken under a microscope and recorded as 0 h. The 6-well plate was placed in an incubator and incubated for 24 hours. After that, the plate was removed, and washed twice with PBS. Images were taken under the microscope at the position points of each well at 0 h, and the migration of cells after 24 hours was recorded.

### Cell apoptosis detection

Flow cytometry with FITC/PI double staining was used to detect cell apoptosis. After different treatments for 24 hours, the cells were treated with trypsin, centrifuged, and washed with cold 1×PBS. Then, the cells were resuspended in 200 μL of binding buffer and mixed with 5 μL of Annexin V + FITC and 5 μL of PI. The mixture was incubated in the dark at room temperature for 15 minutes. Finally, the cells were mixed with 300 μL of binding buffer.

### Cell ferroptosis detection

To determine the intracellular iron concentration, we stained the cells using the Calcein AM staining kit and measured the fluorescence intensity at a wavelength of 494 nm using a fluorescence microplate reader after the incubation period. To determine ferroptosis, we treated the cells with DMSO, ferroptosis inducer erastin (5 uM). Then cell viability was examined by trypan blue staining.

### Statistical analysis

All data followed a normal distribution. Statistical signiﬁcance between groups was determined using one-way ANOVA (Prism 9.0, GraphPad Software). The statistical signiﬁcance was set as *p* < 0.05, *p* ≤ 0.01 and *p* ≤ 0.001 indicate highly significant differences, with smaller *P* values indicating greater significance. Gray quantiﬁcation of Western blot stripes was performed using Image J software to compare protein expression levels.

## Data Availability

All of the data generated during this study were included in this article.
